# Splenic vein thrombosis in cirrhosis of the liver: A rare case

**DOI:** 10.1016/j.amsu.2022.104439

**Published:** 2022-08-18

**Authors:** Arina Mana Sikana, Husin Thamrin

**Affiliations:** a Faculty of Medicine, Universitas Airlangga, Surabaya, East Java, 60132, Indonesia; b Department of Internal Medicine, Division of Gastroentero and Hepatology, General Hospital Dr. Soetomo, Universitas Airlangga, Surabaya, East Java, 60286, Indonesia

**Keywords:** Thrombosis, Portal hypertension, Liver cirrhosis, Liver biopsy, Treatment

## Abstract

Splenic Vein Thrombosis (SpVT) in a young patient with non-hepatitis B and C liver cirrhosis is an infrequent case generating hemorrhagic manifestations. Herein we report a 28-year-old man presenting with hematemesis, melena, and features of liver cirrhosis. Hematemesis, melena, and ascites resolve following a conservative treatment. Abdominal ultrasound confirmed portal hypertension. Serial endoscopy on day 14, 17 and 1-month evaluation showed grade II-III esophageal varices and severe hypertensive portal gastropathy. Abdominal CT scan with contrast within 1 week after discharge revealed thrombus along ± 5.8 cm, splenomegaly with dilated splenic vein, dilatation and tourtosity of the left gastric vein and visualized distal esophageal vein. Liver biopsy 2 months after hospitalization showed hepatocytes with extensive hydropic degeneration with fibrosis (F3).

## Highlights

• Splenic Vein Thrombosis (SpVT) occurring in a young patient is a rare case
• SpVT might occur without relating to hepatitis-B or hepatitis-C infection.
• In this situation, even mostly asymptomatic, gastrointestinal bleeding secondary to esophageal or gastric varices commonly arises.
• Heparinization should be done to increase survival and prevent recurrent thrombosis.
• Immediate splenectomy is strongly recommended in patients with bleeding esophageal varices to prevent massive bleeding.

## 1 Introduction

Splenic Vein Thrombosis (SpVT) is a prevalent case in male patients during their fifth decade of life. Patients commonly complain of abdominal pain, gastrointestinal bleeding, and spleen enlargement [1]. Blood analysis may show thrombocytopenia or pancytopenia [2]. SpVT commonly does not coincide liver cirrhosis and requires advanced imaging modalities, such as venous-phase celiac angiography [3,4].

SpVT results in an elevated localized sinistral portal pressure, also known as left portal hypertension. Most patients present with left portal hypertension with no significant symptoms and normal liver function, but still, gastrointestinal bleeding secondary to esophageal or gastric varices commonly arises. Nonetheless, many patients with peripheral artery SpVT other than gastric varices rarely bleed. Because patients without esophageal varices are asymptomatic, treatment is considered not obligatory along with tight monitoring [3]. Referring to SCARE 2020 Guidelines [5], we report a rare case of a non-hepatitis B and C liver cirrhosis patient developing SpVT.

## 2 Case illustration

A male patient, aged 28, was admitted to of Dr. Soetomo General Hospital with hematemesis and melena. The patient had ascites within the last 6 months accompanied by intermittent abdominal pain. He ever experienced such symptoms 6 years ago. The patient underwent treatment for a week at Sakinah Hospital once and received 3 bags of packed red cell (PRCs), then he was discharged. Three years later, patient was hospitalized twice at Dian Husada Hospital for 6 bags of PRCs transfusion. Abdominal ultrasound revealed liver disease. Patient had a history of chronic pancreatitis.

Vital sign examination showed blood pressure (BP) 100/60 mmHg, pulse 90/minute, respiration rate (RR) 20/minute, oxygen saturation 98% free air, axillary temperature 36,5 °C. Patient weighed 54.5 kg with an abdominal circumference of 95 cm. Physical examination revealed anemic conjunctiva and palms with ascites and hepatosplenomegaly. Laboratory examination on admission showed pancytopenia, normal hemostasis, non-reactive hepatitis B and C viral markers, negative rapid HIV, and negative ADA test (see Table 1). Unfortunately, we didn't obtain anti-HCv examination since it's not covered by national health insurance. The patient underwent conservative treatment with low-salt H2 diet 2100 kcal/day, intravenous furosemide 20 mg BID, lansoprazole pump infusion 6 mg/hour, intravenous cefotaxime 1g TID, Lactulose syrup 30 ml QID, Sucralfate syrup 30 ml per oral TID, Spironolactone 100 mg per oral BID, intravenous tranexamic 500 mg TID, intravenous phytomenadione 10 mg every TID, intravenous octreotide 50 g/hour, and PRC transfusion of PRC 1 bag/day until Hb > 8gr/dL. Continous laboratory marker monitoring was done on day 3, 8 and 12 of hospitalization (See Table 1).

**Table 1:** Laboratory evaluation during hospitalization.

Laboratory		On admission	Day 3	Day 8	Day 12
Hb	g/dL	4.2	8.4	6.8	10.3
WBC	10^3/uL	2.93	1.99	3.17	3.17
RBC	10^6/uL	1.76	3.19	3.89	3.95
HCT	%	13.3	25.9	19.8	30.9
PLT	10^3/UL	76	88	88	63
MCV	fL	75.6	81.2	80	84
MCHC	g/dL	31.6	32.4	31	35
Eosinophill	%	1	1	1	1
Basophill	%	0.3	0.3	0.2	0.1
Neutrophill	%	67.3	60.3	68.2	73.1
Lymphocyte	%	21.5	21.5	21.5	21.5
Monocyte	%	9.9	8	5	4
aPTT	seconds	28.1	28.1		
PPT	seconds	12.8	12.8		
Blood glucose	mg/dL	177	170	156	167
Total bilirubin	mg/dL	0,15	0.95	0.95	
Direct biliruibin	mg/dL	0,55	0.20	0.18	
AST	U/L	23	18	18	
ALT	U/L	31	25	25	
Albumin	g/dL	3.0	3.2		
BUN	mg/dL	8	8	12	
Serum Creatinin	mg/dL	0.64	0.7	0.6	
Electrolycte serum					
Na	mmol/l	133	130	145	
K	mmol/l	3.7	4.0	3.7	
Cl	mmol/l	107	100	102	
AFP	g/dL	0.4			
HBsAg		Non reactive			
Anti HCV		Non reactive			
Rapid HIV		Non reactive			
ADA test		Negative			

ADA: Adenosine Deaminase; AFP: alpha-fetoprotein; ALT: alanine transaminase; aPTT: activated partial thromboplastic time; AST: aspartate transaminase; BUN: blood urea nitrogen; Hb: hemoglobin; HbsAg: Hepatitis B surface antigen; HCT: hematocrite; HCV: hepatitis C virus; HIV: human immunodeficiency virus; K: potassium; MCV: mean corpuscular volume; MCHC: mean corpuscular hemoglobin concentration; Na: sodium; PLT: platelets; PTT: plasma prothrombin time; RBC: red blood cell; WBC: white blood cell; Cl: chloride.

**Fig. 1:**
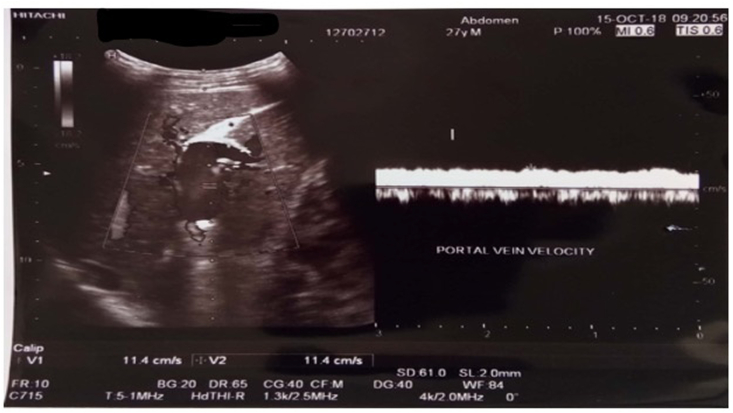

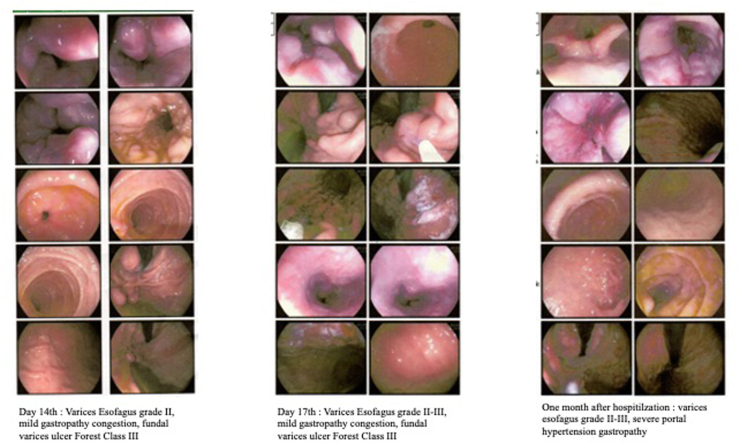

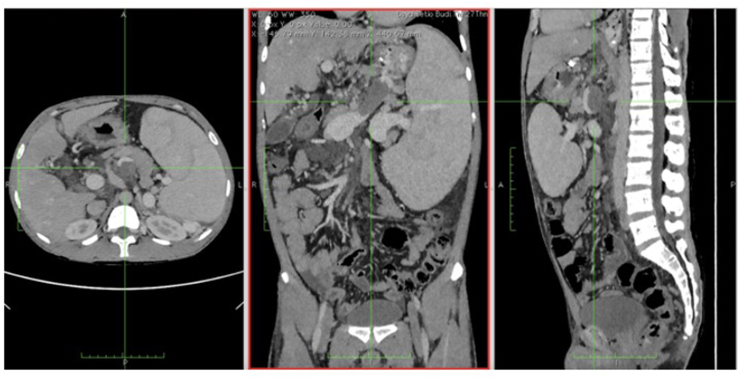
**A.** Abdominal ultrasound showing liver cirrhosis with hypertension portal; **B:** serial esofagogastroduodenoscopy (EGD) on day 14, day 17, and one month after hospitalization; **C:** Abdominal CT with contrast showed thrombus in splenic vein along with splenomegaly.

Abdominal ultrasound on day 7 detected liver cirrhosis with portal hypertension (See Fig. 1A). Esophagogastroduodenoscopy (EGD) evaluation on day 14 and 17 of portrayed grade II-III esophageal varices, mild gastropathy congestion and fundal varices (Forest Class III) (See Fig. 1B). Patient was discharged after eighteen days of treatment. Abdominal CT Scan examination 1 week after hospitalization showed a splenic vein thrombus ± 5.8 cm, splenomegaly with dilated splenic vein, dilatation and tourtosity of the left gastric vein and visualized distal esophageal vein (See Fig. 1C). Endoscopic evaluation one month after hospitalization found esophageal varices II-III and severe portal hypertenesion gastropathy (See Fig. 1B). Liver biopsy was succesfully executed 2 months after hospitalization revealing pieces of liver tissue consisting of 6 portal tracts with disturbed architecture, minimal lymphocytic infiltration, and extensive hydropic degeneration with fibrosis (F3). However, spleen biopy showed no gross pathology and microscopic histopathological of splenic vein thrombosis.

## 3 Discussion

Portal hypertension due to splenic vein thrombosis (SpVT) can induce massive gastrointestinal bleeding from the esophagus or gastric varices and develop hypertensive gastropathy. Acute and chronic pancreatitis, pancreatic pseudocyst, and pancreatic adenocariconma acocompanied 7%–20% of patients with SpVT [1]. Pancreatitis and perivenous inflammation are the most common causes of SpVT [3]. Although more than 45% of SpVT patients with chronic pancreatitis have been reported, many of them are asymptomatic [2].

SpVT can induce local hypertension of the splenic vein and create collateral from the spleen to the fundus. The blood therefore returns to the main portal system via the coronary veins. In some cases, gastric varices are often not associated with esophageal varices except for collateral at the gastroesophageal junction, which is the most common site of bleeding. In other cases, spontaneous bleeding is uncommon. Patients with history of previous pancreatitis are suspicious for having SpVT due to enlarged retroperitoneal lymph nodes located near the splenic artery, above the splenic vein. Other risk factors are history of gastrointestinal bleeding, splenomegaly without portal venous hypertension, cirrhosis or haematological disease and gastric varices [3].

Clinicians shall examine the primary cause of hypercoagulation in any SpVT patient with splenomegaly. Hypercoagulation, both hereditary and acquired, predisposes the patient to arterial or venous thrombosis in brain, extrimity, and intra-abdomen, with venous thromboembolism (VTE) as the most common manifestation. Other disorders caused by hypercoagulation include myeloproliferative syndrome, hyperhomosysteinemia syndrome, and antiphospholipid antibodies (APAs).

Splenic vein obstruction can cause retroperitoneal, pancreatic, and perisplenic lymphadenopathy that leads to vein compression, obstruction, and thrombosis [6].

Ascites is a pathological condition due to accumulation of fluid in the intraperitoneal cavity. Ascites is still the leading complication of cirrhosis within first 10 years, detected in roughly 60% of patients with compensated cirrhosis [7]. Portal hypertension still highly underlies ascites in 75% of the patients despite other pathogenesis explained in established literature [8]. Portal hypertension is induced by increased resistance in the liver, connective tissue, regenerative nodules, vasoconstriction, and thrombus. This condition results in the formation of collateral vein around the portal vein, in the skin, esophagus and stomach. In addition, portal hypertension will also cause splanchnic vein vasodilation which manifesting splenomegaly. Moreover, in the central circulation system, portal hypertension causes systemic vasodilation resulting in effective hypovolemia and activates renin-angiotensin-aldosterone system (RAAS) and vasopressin. This leads to renal vasoconstriction and sodium and water retention. Retention of sodium and water results in the development of refractory ascites. Additionally, this retention also results in increased cardiac output (CO), thereby elevating flow to the portal and exacerbate the existing portal hypertension [9,10] SpVT causes localized left venous hypertension returning splenic venous flow to low-pressure collateral vein whereby preventing blood circulating from the spleen. Flow through the short gastric and/or gastro-epiploic vein dilates the sub-mucosal venous system of the stomach and esophagus. Both will form a thin gastric wall and esophageal varices [6]. Since coronary vein supplies the portal system, gastric varices without esophageal varices highly suggests splenic vein occlusion.

SpVT is diagnosed based on abdominal CT, abdominal angiography, MRI, or ultrasound to distinguish with the differential diagnoses, which are Budd-Chiari and Banti syndrome [11]. Furthermore, anticoagulation issue in isolated SpVT is still an unsolved problem. In acute or subacute mesenteric venous thrombosis, heparinization should be done to increase survival and prevent recurrent thrombosis. Anticoagulants can still be administered for gastrointestinal bleeding as long as the benefit of preventing infarction outweighs the risk of bleeding [1]. Immediate splenectomy is strongly recommended in patients with bleeding esophageal varices to prevent esophageal varices from bleeding massively. Besides, no other available treatment is able to control bleeding [12] Ultimately, splenectomy is the treatment of choice and able to effectively remove collateral outflow [13].

## 4 Conclusion

SpVT is a rare case in patients with non-B and C liver cirrhosis. Bleeding manifestations are emergencies in need of comprehensive management. Proper imaging i.e abdominal CT with contrast helps diagnose SpVT. Serial EGD evaluation is required to evaluate complications of esophageal varices that possibly reappear at any time.

## Ethical approval

The approval has been given by the patient.

## Sources of funding

No external source of funding.

## Author contributions

Arina Mana Sikana ê case illustration, interpretation, manuscript arrangement, final editing.

Husin Thamrin ê case illustration, supervision, final editing.

## Trial registry number

1.Name of the registry:

2.Unique Identifying number or registration ID:

3.Hyperlink to your specific registration (must be publicly accessible and will be checked):

## Guarantor

Both author are the guarantor of this work.

## Consent

The patient has signed an informed consent.

## Funding

The author(s) received no financial support for the research, authorship, and/or publication of this article.

## Declaration of competing interest

All authors declare no conflict of interest.
